# Strangulation and Necrosis of an Epiploic Appendage of the Sigmoid Colon in a Right Inguinal Hernia

**DOI:** 10.1155/2013/890234

**Published:** 2013-09-11

**Authors:** Yuri N. Shiryajev, Anna V. Glebova, Marina V. Chalenko

**Affiliations:** ^1^Department of Faculty Surgery named after Professor A.A. Rusanov, Saint Petersburg State Pediatric Medical University, Litovskaya Street 2, Saint Petersburg 194100, Russia; ^2^Sixth Department of Surgery, Mariinsky Hospital, Saint Petersburg, Russia

## Abstract

An epiploic appendage of the sigmoid colon is considered to be an unusual type of inguinal hernia content. The strangulation of a sigmoid colon appendage into a right inguinal hernia is exclusively rare. We present a case of an 81-year-old female patient with severe cardiovascular comorbidities who was urgently admitted after an episode of strangulation and subsequent spontaneous reduction of a right inguinal hernia. The condition of the patient was stable, and an urgent operation was not indicated for three days after admission. However, we had to operate because the hernia strangulation recurred. In the hernia sac, a free fatty body (a separated and saponified epiploic appendage of the colon) and a strangulated epiploic appendage of dolichosigmoid, with signs of necrosis, were found. Removal of the free fatty body and necrotic epiploic appendage and subsequent anterior-wall inguinal hernioplasty were successfully performed. In the world literature, this case may be the first report of a sigmoid epiploic appendage strangulation in a right inguinal hernia that is well documented by photography.

## 1. Introduction

It is well known that the most common content of a strangulated inguinal hernia is a small bowel loop or part of the major omentum [1]. Certain parts of the colon, urinary bladder, vermiform appendix, Meckel's diverticulum, uterine tube and ovary, and other tissues [1] may be observed in a strangulated groin hernia much less frequently. *Epiploic appendages* of the colon are considered to be one of the rarest types of such hernia content [2, 3].

This clinical situation (the strangulation of an epiploic appendage in an inguinal hernia) was described, independently and for the first time, by von Bruns and Schweinburg in 1906, as noted in a review by Hunt [4]. A similar condition (the intrahernial torsion of an epiploic appendage) was first presented for a femoral hernia by Riedel in 1905 and for an inguinal hernia separately by Serve and Muscatello in 1906 [4]. The number of published cases then rapidly increased, and by the second decade of the 20th century, approximately 22 observations of intrahernial complications (10 torsion and 12 strangulation cases) involving epiploic appendages were reported in the world literature [4]. Currently, such cases are still being published [2, 3, 5, 6], particularly with the addition of new imaging modalities for diagnosis [7, 8]. The overwhelming majority of publications present the strangulation of a sigmoid epiploic appendage in a left inguinal hernia [2–7, 9]. We would like to present an exclusively rare case of the strangulation and necrosis of an epiploic appendage of the sigmoid colon in a *right* inguinal hernia (RIH).

## 2. Case Presentation

A female 81-year-old patient, L., was urgently admitted and presented with pain in the right inguinal area, a soft mass in that area, nausea, and two instances of vomiting bile. These complaints had persisted for approximately 24 hours, without improvement of the patient's condition. The patient's RIH history was about 5 years long, with rare episodes of strangulation and subsequent self-reduction of the hernia. The patient had serious concomitant diseases (ischemic heart disease, cardiosclerosis, severe arterial hypertension, stable atrial fibrillation, a small left hydrothorax, and chronic heart failure (stage IIb)). The day before admission, she was discharged from the cardiology department, where she was treated for a heart failure for 13 days, with clear improvement. For a long time, the patient had complained of constipation and used herbal laxatives and enemas. 

Considering the clinical and anamnestic data, a diagnosis of RIH with strangulation and subsequent spontaneous reduction was made. There were no indications for urgent surgery, and the patient was left for clinical observation. For three days, the condition of the patient was stable. However, on the fourth day, after straining, recurrent strangulation of the hernia occurred. An urgent operation was indicated.

**Figure 1:**
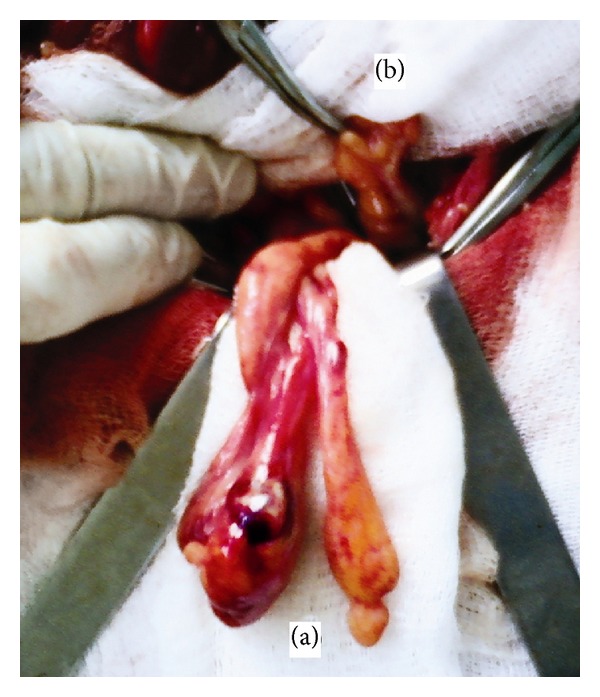
A view of the operating field in the right inguinal area: a two-tailed epiploic appendage of the sigmoid colon with the signs of necrosis (a) and a normal vermiform appendix (b).

**Figure 2:**
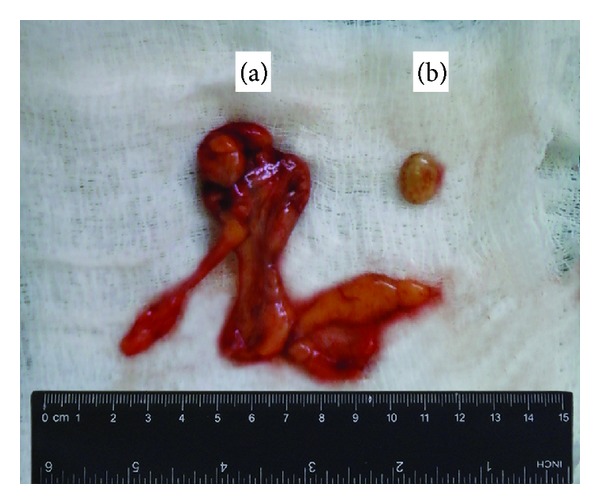
The removed specimen: an epiploic appendage (a) and a free fatty body (b) found in the hernia sac.

Under general anesthesia, the right inguinal canal was opened using a standard approach. The hernia sac (its size was 12 × 8 × 7 cm) was opened, and a certain amount of yellow hernia water was discharged. A free fatty body of ovoid form (15 × 12 mm) was found in the sac and removed. An epiploic appendage of the sigmoid colon, 10 × 3 × 2 cm in size, with signs of necrosis, was present (Figure 1(a)). In the depth of the wound, the sigmoid colon (dolichosigmoid), partially located in a right iliac fossa, was clearly observed. The normal vermiform appendix was present in the upper part of the wound (Figure 1(b)). The epiploic appendage was excised (the specimen is presented in Figure 2(a)), and the hernia sac was resected. The anterior wall of the right inguinal canal was augmented using Girard hernioplasty with Kimbarovskiy's modification [10].

The postoperative course was uneventful. The wound healed by primary intention, and the patient was discharged in a satisfactory condition on the seventh postoperative day.

Upon histological examination of the removed appendage, fatty tissue with wide fields of necrosis was found. The free fatty body was not examined for unknown reasons. Upon checkup in the clinic 6 weeks later, the patient was alive and well, but her constipation still existed, and she continued to use herbal laxatives and enemas. The RIH did not recur. Ten weeks after the operation, the patient died due to cardiac arrest. An autopsy was not performed.

## 3. Discussion

Epiploic appendages of the colon, either strangulated or volvulized, can be found in the hernia sacs of inguinal hernias [4, 9]. Although this variant of hernia content is rare [2, 3, 5], as early as 1919, Hunt (Mayo Clinic, Rochester, USA) surveyed 22 cases, in which an epiploic appendage was found in the hernia sac of a groin hernia, from the literature and added two cases from the Mayo Clinic [4]. Recently, certain case reports on this topic have been published [2, 3, 5–7]. A literature analysis shows that the overwhelming majority of the cases are reports of the strangulation (or simple localization) of a sigmoid epiploic appendage in a left groin hernia. This phenomenon is associated with the highest rate of torsion and primary inflammation of appendages with precisely this localization. 

However, a long sigmoid colon can reach the right ilioinguinal area, and this anatomical phenomenon can lead to unexpected surgical findings. The classical finding is epiploic appendagitis (typically due to torsion of the appendage), which can mimic acute appendicitis in that anatomical situation [4, 11, 12]. During the last decade, certain unique versions of complicated RIH have been published: the strangulation and necrosis of the dolichosigmoid [13]; the combination of a dolichosigmoid volvulus with its strangulation in a RIH [14]; and sigmoid colon cancer, complicated by an obstruction and/or paracolic abscess, with the tumor located in the hernia sac of a RIH [15, 16]. These research studies cannot be considered to be “purely” casuistic, even though the studies are very interesting and significant in this aspect; particularly rare cases, excellent illustrations, and optimal surgical tactics also make these publications very valuable. A wide range of recent actual problems are presented in this section: the possibility of preoperative diagnosis using modern imaging techniques; the choice of the operation extent; and indications for prosthetic groin hernioplasty in complicated hernias. In some above mentioned cases, the surgeons had to convert the approach to median laparotomy to allow the correct surgical incision for an obstructive sigmoid colon resection. If these complications had a left-sided location, it would have been justified to decrease the traumatism of the procedure using herniolaparotomy or a left alternating (McBurney's-like) approach after extension of the skin incision. Fortunately, in our case, the necrotic changes were limited to the epiploic appendage and were not extended to the bowel wall. We were able to correctly perform an operation using the primary right inguinal approach. 

We consider the recent necrosis of the epiploic appendage to be the result of previous hernia strangulation. In the last episode of strangulation, which occurred in the hospital, the patient was transferred to the operating room very promptly, and we think that the observed necrotic changes in the appendage could not have developed during such a short time period (less than 2 hours). The recurrent strangulation was the indication for urgent surgery, and the surgery's extent (the removal of the free fatty body and resection of the necrotized epiploic appendage, with subsequent anterior-wall hernioplasty) was the logical implication of the operative findings.

To the best of our knowledge, our report may be the first in the literature, published at the PubMed era and well documented by photography, when the epiploic appendage of the sigmoid colon was the content of a strangulated RIH. However, it is not the first such case in general. A thorough analysis of the old literature allowed us to find a few similar cases [17–20], and in all these cases an involved epiploic appendage removal and a hernioplasty were performed as necessary parts of an operation. A distinctive feature of our case is the presence of both a necrotic epiploic appendage and a free fatty body in a hernia sac. The free fatty body (Figure 2(b)) can be considered as a sequela of prior episodes of hernia strangulation or intrahernial torsion of an epiploic appendage. 

Finally, there are a few words about the chosen type of the hernioplasty. Outside Russia, modifications of the anterior wall inguinal herniorrhaphy (antefunicular or anteligamental) were abandoned many years ago. In certain textbooks on surgery and herniology, such versions of hernioplasty are just not described. In our country, due to the historically established traditions, an anterior wall hernioplasty is still used. Small indirect hernias in young males (to avoid the traumatic mobilization of the spermatic cord), strangulated indirect hernias in old and debilitated patients, and female hernias are the most common indications for this type of herniorrhaphy. We augmented the anterior wall of the inguinal canal in the presented case to decrease traumatism and duration of the procedure—it was important in a patient with severe cardiologic conditions. On the other hand, this type of hernioplasty was sufficiently reliable and strong. We consider our surgical tactics to be case-oriented and warranted.

## Abbreviations

RIH: Right inguinal hernia.
